# Disruption of Genes Encoding eIF4E Binding Proteins-1 And -2 Does Not Alter Basal or Sepsis-Induced Changes in Skeletal Muscle Protein Synthesis in Male or Female Mice

**DOI:** 10.1371/journal.pone.0099582

**Published:** 2014-06-19

**Authors:** Jennifer L. Steiner, Anne M. Pruznak, Gina Deiter, Maithili Navaratnarajah, Lydia Kutzler, Scot R. Kimball, Charles H. Lang

**Affiliations:** Department of Cellular and Molecular Physiology, and Surgery, Penn State College of Medicine, Hershey, Pennsylvania, United States of America; University of Florida College of Medicine, United States of America

## Abstract

Sepsis decreases skeletal muscle protein synthesis in part by impairing mTOR activity and the subsequent phosphorylation of 4E-BP1 and S6K1 thereby controlling translation initiation; however, the relative importance of changes in these two downstream substrates is unknown. The role of 4E-BP1 (and -BP2) in regulating muscle protein synthesis was assessed in wild-type (WT) and 4E-BP1/BP2 double knockout (DKO) male mice under basal conditions and in response to sepsis. At 12 months of age, body weight, lean body mass and energy expenditure did not differ between WT and DKO mice. Moreover, in vivo rates of protein synthesis in gastrocnemius, heart and liver did not differ between DKO and WT mice. Sepsis decreased skeletal muscle protein synthesis and S6K1 phosphorylation in WT and DKO male mice to a similar extent. Sepsis only decreased 4E-BP1 phosphorylation in WT mice as no 4E-BP1/BP2 protein was detected in muscle from DKO mice. Sepsis decreased the binding of eIF4G to eIF4E in WT mice; however, eIF4E•eIF4G binding was not altered in DKO mice under either basal or septic conditions. A comparable sepsis-induced increase in eIF4B phosphorylation was seen in both WT and DKO mice. eEF2 phosphorylation was similarly increased in muscle from WT septic mice and both control and septic DKO mice, compared to WT control values. The sepsis-induced increase in muscle MuRF1 and atrogin-1 (markers of proteolysis) as well as TNFα and IL-6 (inflammatory cytokines) mRNA was greater in DKO than WT mice. The sepsis-induced decrease in myocardial and hepatic protein synthesis did not differ between WT and DKO mice. These data suggest overall basal protein balance and synthesis is maintained in muscle of mice lacking both 4E-BP1/BP2 and that sepsis-induced changes in mTOR signaling may be mediated by a down-stream mechanism independent of 4E-BP1 phosphorylation and eIF4E•eIF4G binding.

## Introduction

The loss of skeletal muscle and lean body mass (LBM) is a central feature of septic and critically ill patients [1], [2]. This cachexic state is noteworthy as it is associated with increased complications and delayed recovery from sepsis [3], [4] as well as increased mortality [5], [6]. In the post-absorptive state, the sepsis-induced decrease in muscle mass is caused by both increased rates of proteolysis and decreased rates of protein synthesis [7], [8]. Furthermore, infection in general and bacterial products and inflammatory mediators in particular also produce an anabolic resistance to hormonal- and nutrient-stimulated increases in protein synthesis [9]–[11].

The cellular mechanism responsible for the sepsis-induced decrease in skeletal muscle protein synthesis results at least in part from impaired kinase activity of mTOR (mammalian target of rapamycin), as evidenced by the reduced phosphorylation of two down-stream substrates – eukaryotic initiation factor (eIF) 4E binding protein (4E-BP1) and the 70 kDa ribosomal protein S6 kinase (S6K)-1 [8], [12], [13]. Furthermore, a comparable reduction in protein synthesis and mTOR signal transduction is observed under in vitro conditions in differentiated myotubes incubated with endotoxin (lipopolysaccharide; LPS) and interferon (IFN)-γ [14], [15]. The ability of sepsis and LPS from the cell wall of gram-negative bacteria to stimulate the local and systemic production of inflammatory mediators [tumor necrosis factor-alpha (TNFα), interleukin (IL)-6, nitric oxide synthase (NOS)-2] and/or reduce anabolic stimuli [e.g., insulin-like growth factor (IGF)-I] appears central to mTOR inhibition [9], [15]–[19].

As a cellular sensor integrating stimulatory signals from nutrients and growth factors as well as inhibitory inputs from various stressors, mTOR is central to the regulation of diverse metabolic pathways [8], [20]. The canonical mTOR signaling pathway suggests that changes in mTOR kinase activity should coordinately regulate both 4E-BP1 and S6K1 [21], but the overall importance of each protein in regulating muscle protein synthesis in vivo has not been investigated. We addressed this question by using mice globally deficient in both 4E-BP1 and 4E-BP2 [22] so as to minimize any potential compensatory change in the latter isoform complicating data interpretation. These two proteins, along with 4E-BP3 which is of low abundance in muscle [23], comprise the 4E-BP family which is important in the control of mRNA translation [24], [25]. Under catabolic conditions, nonphosphorylated 4E-BP functions as an inhibitor of cap-dependent translation initiation by binding to eIF4E and preventing the interaction of the latter protein with eIF4G [26], [27]. Using 4E-BP1/BP2 double knockout (DKO) mice and their wild-type (WT) littermates, we tested the hypothesis that deletion of 4E-BP1/BP2 would increase basal skeletal muscle protein synthesis and ameliorate the decrement in synthesis observed after induction of sepsis (a catabolic stressor), thereby demonstrating the central importance of eIF4E binding proteins in regulating muscle protein balance under in vivo conditions.

## Methods

### Animal Protocols

All mice were viral antibody free and housed under controlled environmental conditions (12∶12 light:dark). Unless noted, mice were housed 3–4 per cage (solid-bottom) with corn cob bedding. Animals were provided Teklad Global 2019 (calories from protein (23%), fat (22%) and carbohydrate (55%); Harlan Teklad, Boston, MA) and water ad libitum. All breeding and experimental protocols were approved by the Institutional Animal Care and Use Committee of The Pennsylvania State University College of Medicine and adhered to the National Institutes of Health (NIH) guidelines for the use of experimental animals.

*Eif4ebp1* and *Eif4ebp2* mutant mice were generated as previously described [22]. Congenic BALB/c *Eif4ebp1^−/−^* and *Eif4ebp2^−/−^* mice were obtained from Dr. Nahum Sonenberg (McGill University) by backcrossing the original knockout strains to inbred BALB/c mice from Charles River Breeding Laboratories (Cambridge, MA). BALB/c *Eif4ebp1* and *Eif4ebp2* heterozygous mice, genotyped at 4 wks of age, were then bred between 8–10 wks of age to obtain BALB/c *Eif4ebp1; Eif4ebp2* double knockout (DKO) mice.

### Induction of Sepsis

Polymicrobial peritonitis was produced by cecal ligation and puncture (CLP), as described and characterized previously [28]–[30]. Briefly, mice were anesthetized using isoflurane (Abbott Laboratories, North Chicago, IL). Hair on the abdomen was clipped and the skin prepared with povidone-iodine, which was followed by a midline incision (1.5 cm) below the diaphragm. The cecum was isolated, ligated, punctured twice with a 25-gauge needle, and a small amount of cecal material extruded to ensure patency. The cecum was returned to the abdomen, the muscle incision closed with 6-0 surgical suture (Ethicon, Inc., Somerville, NJ), and metal wound clips were used to close the skin incision. Before suturing the skin, 2 to 3 drops of lidocaine (Abbott Laboratories) were administered to the wound for analgesia. All mice were housed individually after surgery and each received 1 mL of warmed (37°C) 0.9% sterile saline containing 0.05 mg/kg of buprenorphine (Reckitt Benckiser Pharmaceuticals Inc, Richmond, VA) administered subcutaneously every 12 h. Sham controls were subjected to the same surgical laparotomy and cecal isolation, but the cecum was neither ligated nor punctured. Any mouse determined to be moribund was anesthetized and killed instead of waiting for spontaneous death [31]. Before surgery, animals had unrestricted access to food and water. As septic mice consume little or no food during the first 24 h post-CLP, food was withheld from all mice so metabolic differences between control and septic mice could be assessed independent of differences in caloric intake. Control and septic mice were anesthetized with isoflurane 24-h after induction of sepsis by CLP and tissues collected.

### Body Composition and Metabolic Rate

Longitudinal changes in body composition were monitored non-invasively in conscious animals using a ^1^H-NMR analyzer (Bruker LF90 Proton-NMR Minispec: Bruker Optics, Woodlands, TX) for rapid measurement of total body lean and fat mass [28], [32]. Indirect calorimetry measurements were obtained in WT and DKO mice at 12 months of age (TSE Systems Inc, Midland, MI) and used to measure CO_2_ and O_2_ levels, as previously described [33]. Each cage was equipped with a sensor frame (MoTil2, TSE Systems) containing infrared beams for determining spontaneous locomotor activity. Energy expenditure was calculated over a continuous 24-h period (kcal/h/kg body weight) after 48 h of acclimation with ad libitum access to food and water throughout. Respiratory exchange ratio (RER) was calculated as the ratio whole-body carbon dioxide production (VCO_2_)/oxygen consumption (VO_2_).

### In vivo Protein Synthesis

The in vivo rate of protein synthesis in the gastrocnemius (hereafter referred to as muscle), heart and liver was determined in WT and DKO mice under control conditions and 24 h after sepsis induced by CLP. Protein synthesis was determined using the flooding-dose technique, as previously described [34]. Mice were injected intraperitoneally with [^3^H]-L-phenylalanine (150 mM, 30 µCi/ml; 1 ml/100 g body wt) and blood was collected 15 min later. Thereafter, mice were anesthetized with isoflurane and tissues were rapidly excised. A portion of each tissue was freeze-clamped, stored at −70°C, and processed as previously described [34]. The plasma phenylalanine concentration and radioactivity was measured by HPLC analysis of supernatant from TCA extracts of plasma. The rate of protein synthesis was calculated by dividing the amount of radioactivity incorporated into protein by the plasma phenylalanine-specific radioactivity. In addition, samples of fresh tissue were homogenized for Western blot analysis and another piece of tissue processed for qRT-PCR.

### Western Blot Analysis

Fresh tissue was homogenized (Kinematic Polytron; Brinkmann, Westbury, NY) in ice-cold homogenization buffer consisting of (in mmol/L): 20 HEPES (pH 7.4), 2 EGTA, 50 sodium fluoride, 100 potassium chloride, 0.2 EDTA, 50 β-glycerophosphate, 1 DTT, 0.1 phenylmethane-sulphonylfluoride, 1 benzamidine, and 0.5 sodium vanadate. The protein concentration was quantified using a Pierce BCA protein assay kit (Thermo Scientific, Rockford, IL) and equal amounts of total protein per sample were subjected to standard SDS-PAGE. Specifically, Western blot analysis was performed for total and phosphorylated (T37/46) 4E-BP1 (Bethyl Laboratories, Montgomery, TX), total 4E-BP2, total and phosphorylated (T389) S6K1, total and phosphorylated (S422) eIF4B, total and phosphorylated (T56) eukaryotic elongation factor (eEF)-2 (Dr. C. Proud; Southampton, UK), total programmed cell death protein 4 (PDCD4) and tubulin. All antibodies were from Cell Signaling Technology (Beverly, MA), unless indicated above. To assess binding of eIF4G with eIF4E, the latter protein was immune-precipitated from aliquots of supernatants using an anti-eIF4E monoclonal antibody (Dr. Kimball; Hershey, PA), and the antibody-antigen complexes were collected using magnetic beads, subjected to SDS-PAGE and quantified as above. Blots were developed with enhanced chemiluminescence Western blotting reagents (Supersignal Pico, Pierce Chemical, Rockford, IL), according to detailed methods provided previously [12], [13], [28]. Dried blots were exposed to x-ray film to achieve a signal within the linear range and film was then scanned (Microtek ScanMaker IV; Cerritos, CA) and quantified using Scion Image 3b software (Scion Corporation, Frederick, MD).

### RNA Extraction and Real-time Quantitative PCR

Total RNA was extracted using Tri-reagent (Molecular Research Center, Inc., Cincinnati, OH) and RNeasy mini kit (Qiagen, Valencia, CA) following manufacturers’ protocols and previous reports [14], [28], [32]. Briefly, tissue was homogenized in Tri-reagent followed by phenol/chloroform extraction according to the manufacturer’s instruction. An equal volume of 70% ethanol was added to the aqueous phase and the mixture was loaded on a Qiagen mini-spin column. The Qiagen mini-kit protocol was followed from this step onwards including the on-column DNase I treatment to remove residual DNA contamination. RNA was eluted from the column with RNase-free water and an aliquot was used for quantitation (NanoDrop 2000, Thermo Fisher Scientific, Waltham, MA). Quality of the RNA was analyzed on a 1% agarose gel. Total RNA (1 µg) was reversed transcribed to cDNA using superscript III reverse transcriptase (Invitrogen, Carlsbad, CA) in a total reaction volume of 20 µl following instructions from the manufacturer. Real-time quantitative PCR was performed on 1–2 µl of the reversed transcribed reaction mix in a StepOnePlus system using TaqMan gene expression assays (Applied Biosystems, Foster City, CA) for: atrogin 1 (F-box protein 32; NM_026346.2), muscle RING-finger 1 (MuRF1; NM_001039048.2), interleukin (IL)-6 (NM_031168.1), tumor necrosis factor (TNF)-α (NM_013693.2), and ribosomal protein L32 (NM_172086.2). The comparative quantitation method 2^−ΔΔCt^ was used in presenting gene expression of target genes in reference to the endogenous control.

### Statistics

Data for each condition are summarized as means ± standard error of the mean (SEM) where the number of mice per treatment is indicated in the legend to the figure or table. Statistical evaluation of the data was performed using 3-way (genotype×sepsis×sex) ANOVA with post-hoc Student-Neuman-Keuls test when the interaction was significant. For some endpoints, a 2-way ANOVA with repeated measures was performed. Differences between groups were considered significant at *P*<0.05. GraphPad Prism version 5.0 (GraphPad software, La Jolla, CA) was used for statistical analysis.

## Results

### Body and Tissue Weight, Body Composition and Energy Expenditure

**Figure 1:**
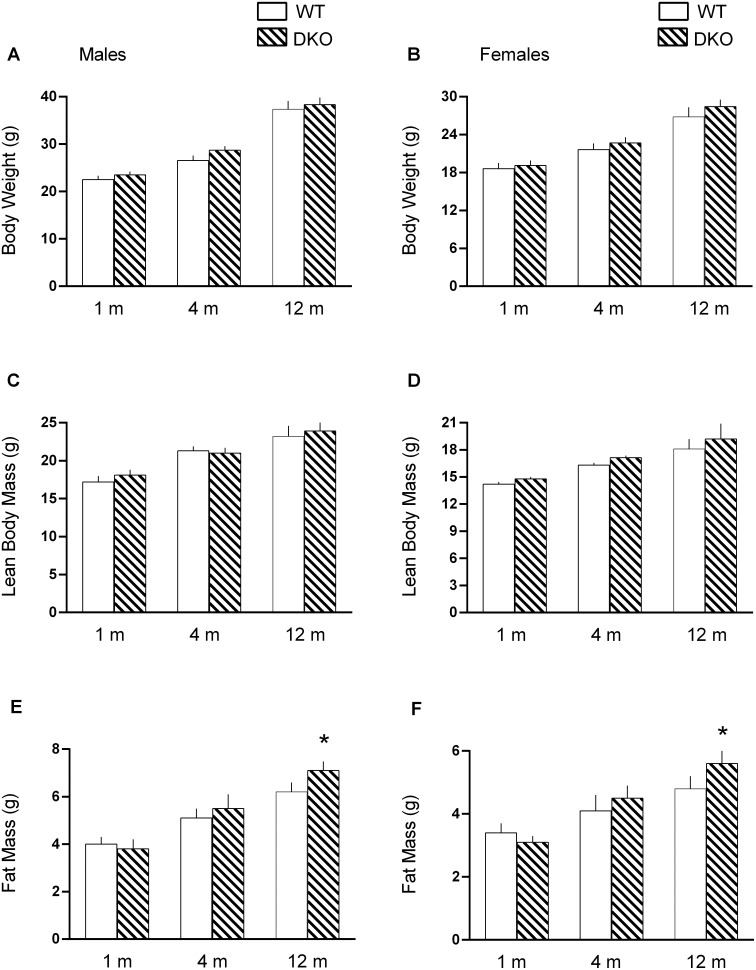
Body weight and body composition in WT and 4E-BP1/BP2 DKO mice. Values are means ± SEM; n = 9–12 per group. *P<0.05, compared to time-matched WT value. There was a significant (*P*<0.05) increase in all parameters 1 month (m) to 12 months of age, regardless of genotype or sex.

Age-induced changes in body weight and composition in WT and DKO mice are presented in Figure 1. As expected, the absolute whole body mass as well as the LBM and fat mass all increased from 1 month to 12 months of age (*P*<0.05, ANOVA repeated measures) for both male and female WT and DKO mice. Body weight and LBM did not differ between WT and DKO at any time point examined for either male (Figure 1A and 1C, respectively) or female (Figure 1B and 1D, respectively) mice; however, fat mass was increased in DKO at 12 months of age in both male and female mice (Figure 1E and 1F).

**Figure 2:**
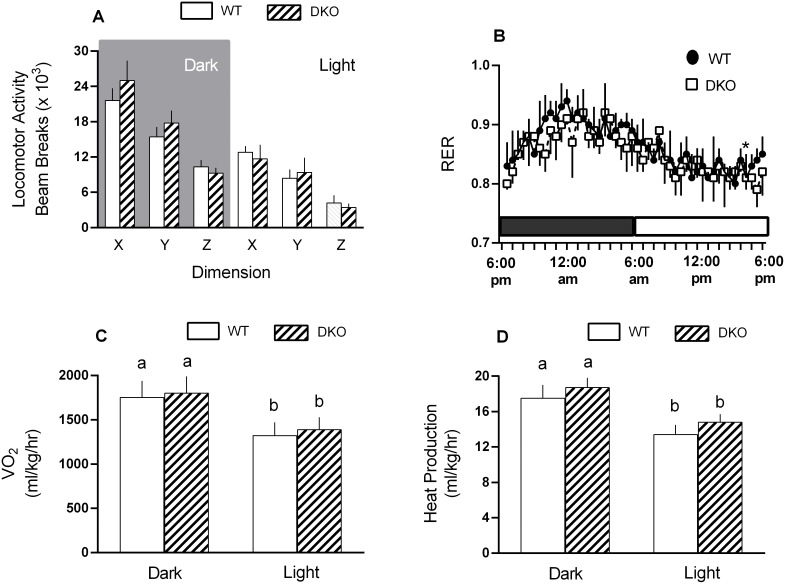
Indirect calorimetry in WT and 4E-BP1/BP2 DKO mice during the light and dark cycle. Spontaneous locomotor activity (panel A) was measured using infrared sensor pairs arranged in strips for horizontal (x, y level) and vertical (z level, rearing) activity; respiratory exchange ratio (RER; panel B), oxygen consumption (VO_2_; panel C) and heat production (panel D) were assessed by indirect calorimetry over a 24-h period after a 48 h acclimatization period. Values are means ± SEM; n = 9–12 per group. For all bar graphs, values having a different superscript letter (a versus b) are statistically different (*P*<0.05); values with the same letter are not significantly different. While there were no significant differences between WT and DKO for any endpoint assessed, both groups had significantly (*P*<0.05) lower levels of activity, RER, VO_2_ and heat production during the light phase compared to the dark phase, but this is not indicated on the figure.

The change in activity, resting energy ratio (RER), oxygen consumption (VO_2_) and heat production was determined for male WT and DKO mice during the light and dark cycle. The level of locomotor activity did not differ between WT and DKO mice during either the dark or light cycle, but the activity level for both groups was significantly (*P*<0.05) lower in the light versus dark phase (Figure 2A). Likewise, there was no genotype difference for RER (Figure 2B), VO_2_ (Figure 2C), VCO_2_ (data not shown) or heat production (Figure 2D); however, for each parameter, values were significantly (*P*<0.05) higher during the dark phase than light phase.

**Figure 3:**
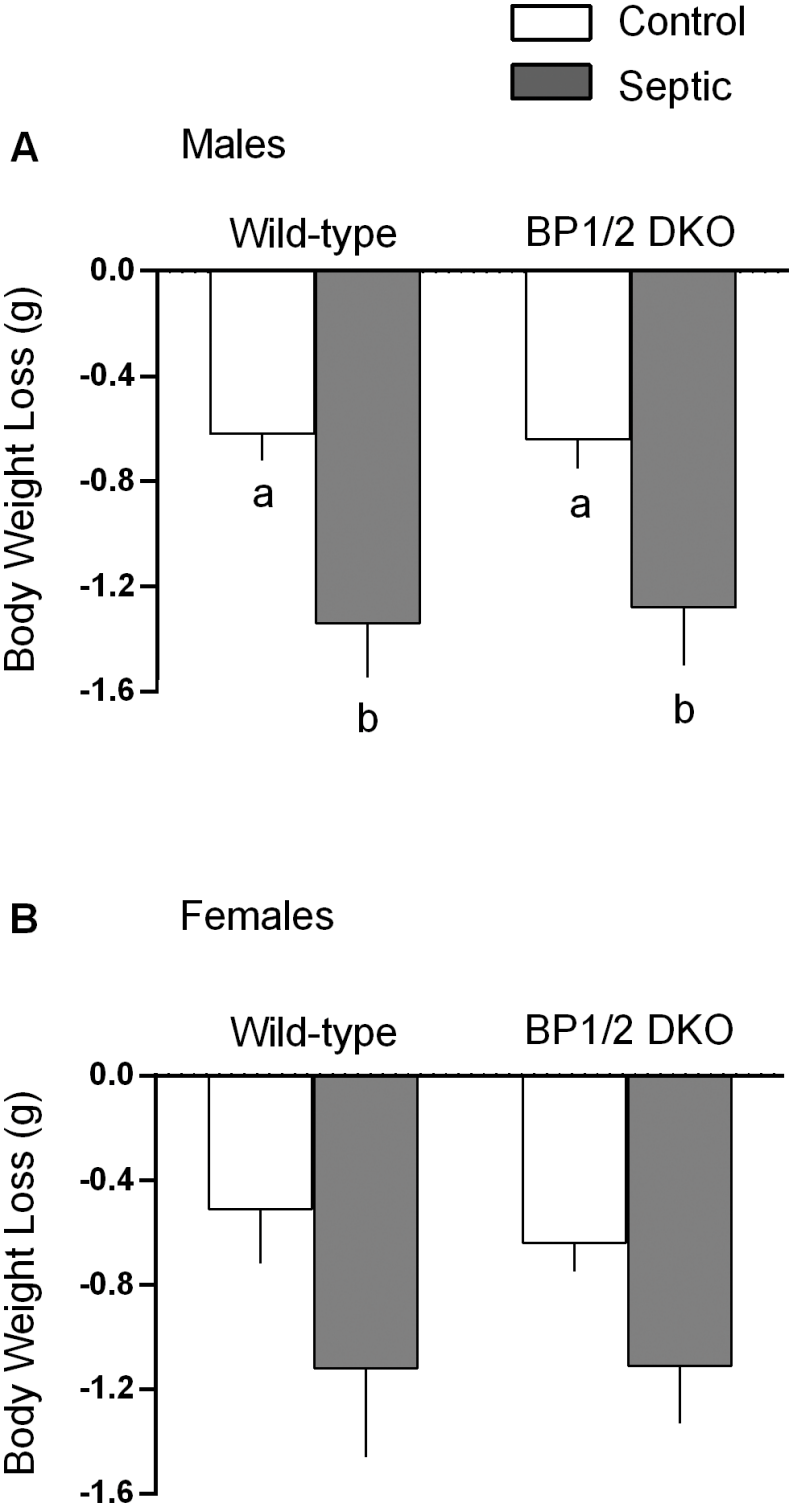
Sepsis-induced body weight loss in male and female WT and 4E-BP1/BP2 DKO mice. Values are means ± SEM; n = 9–12 per group. For all bar graphs, values having a different superscript letter (a versus b) are statistically different (*P*<0.05); values with the same letter are not significantly different.

In male mice, regardless of genotype, the loss of body weight was greater in septic than nonseptic control mice (Figure 3A). The sepsis-induced decrease in body weight did not differ between WT and DKO male mice. A similar trend was seen in female mice, but the sepsis-induced changes failed to achieve statistical significance (Figure 3B).

The data presented in Table 1 indicate there was no sepsis-induced difference in the absolute weight (grams) for the gastrocnemius, quadriceps and heart between WT and DKO mice. However, there was a sex effect on absolute tissue weight, with gastrocnemius, quadriceps and heart from female mice weighing less than the same tissue in male mice. This sex difference was not evident when data were normalized to the animal’s individual body weight (e.g., % BW). There was also no difference in the organ weight for either the liver or kidney between the four groups of mice for either sex (data not shown).

**Table 1:** Skeletal muscle and heart weights of male and female WT and 4E-BP1/BP2 DKO mice under control and septic conditions.

	Males				Females			
	WT		DKO		WT		DKO	
	Control	Septic	Control	Septic	Control	Septic	Control	Septic
**Absolute Weight, mg**								
Gastroc	148	155	161	162	116	115	123	122
	±5	±4	±5	±4	±4	±3	±5	±9
Quad	255	271	263	271	204	192	207	207
	±7	±11	±7	±11	±7	±4	±4	±9
Heart	196	193	188	203	125	131	137	140
	±9	±6	±5	±6	±5	±6	±4	±4
**% Body Weight**								
Gsatroc	0.39	0.4	0.44	0.44	0.43	0.44	0.41	0.41
	±0.03	±0.04	±0.03	±0.07	±0.03	±0.04	±0.03	±0.03
Quad	0.68	0.71	0.72	0.72	0.75	0.71	0.77	0.71
	±0.03	±0.03	±0.05	±0.04	±0.02	±0.03	±0.04	±0.06
Heart	0.51	0.51	0.51	0.54	0.47	0.49	0.51	0.48
	±0.05	±0.04	±0.04	±0.06	±0.04	±0.06	±0.03	±0.02

Values are means ± SEM; n = 9–12 per group. Gastroc = gastrocnemius; Quad = quadriceps. For each tissue, 3-way ANOVA indicated no genotype or sepsis effect, but the absolute weight of each tissue was significantly (*P*<0.05) lower in female compared to male mice. This sex difference was lost when data were normalized to body weight.

### Tissue Protein Synthesis and mTOR Signaling

**Figure 4:**
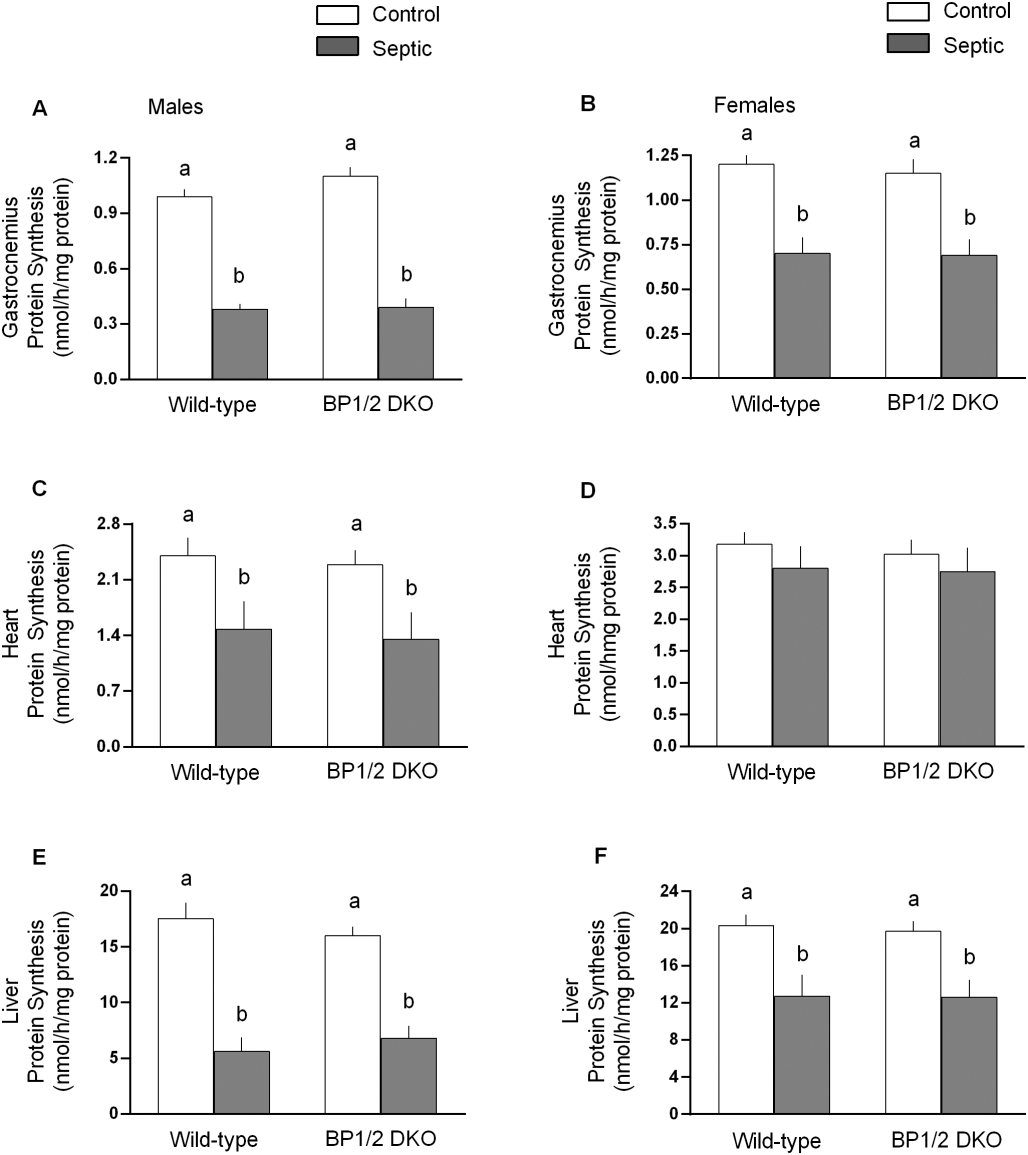
In vivo-determined rates of protein synthesis in gastrocnemius, heart and liver of male and female WT and 4E-BP1/BP2 DKO mice. Values are means ± SEM; n = 9–12 per group. For all bar graphs, values having a different superscript letter (a versus b) are statistically different (*P*<0.05); values with the same letter are not significantly different. For each tissue, female mice had a smaller sepsis-induced decrease in protein synthesis (*P*<0.05).

Contrary to expectations, there was no difference in the rate of protein synthesis between WT and DKO mice under nonseptic control conditions for any tissue examined for either male or female mice (Figure 4). Sepsis decreased protein synthesis in gastrocnemius (60%), heart (38%) and liver (70%) in WT male mice (Figure 4A, 4C and 4E, respectively). The sepsis-induced decrease in tissue protein synthesis did not differ between male DKO and WT mice. Similarly, sepsis decreased protein synthesis in the quadriceps of male WT and DKO mice to the same extent (data not shown). Female WT and DKO mice also showed a comparable sepsis-induced decrease in protein synthesis in gastrocnemius (Figure 4B) and liver (Figure 4F). However, there was a gender interaction in female mice, compared to their male counterparts, indicating the sepsis-induced decrease in skeletal muscle and liver was statistically smaller than in males. Finally, in contrast to the response seen in male mice, sepsis did not alter cardiac protein synthesis in female WT or DKO mice (Figure 4D).

**Figure 5:**
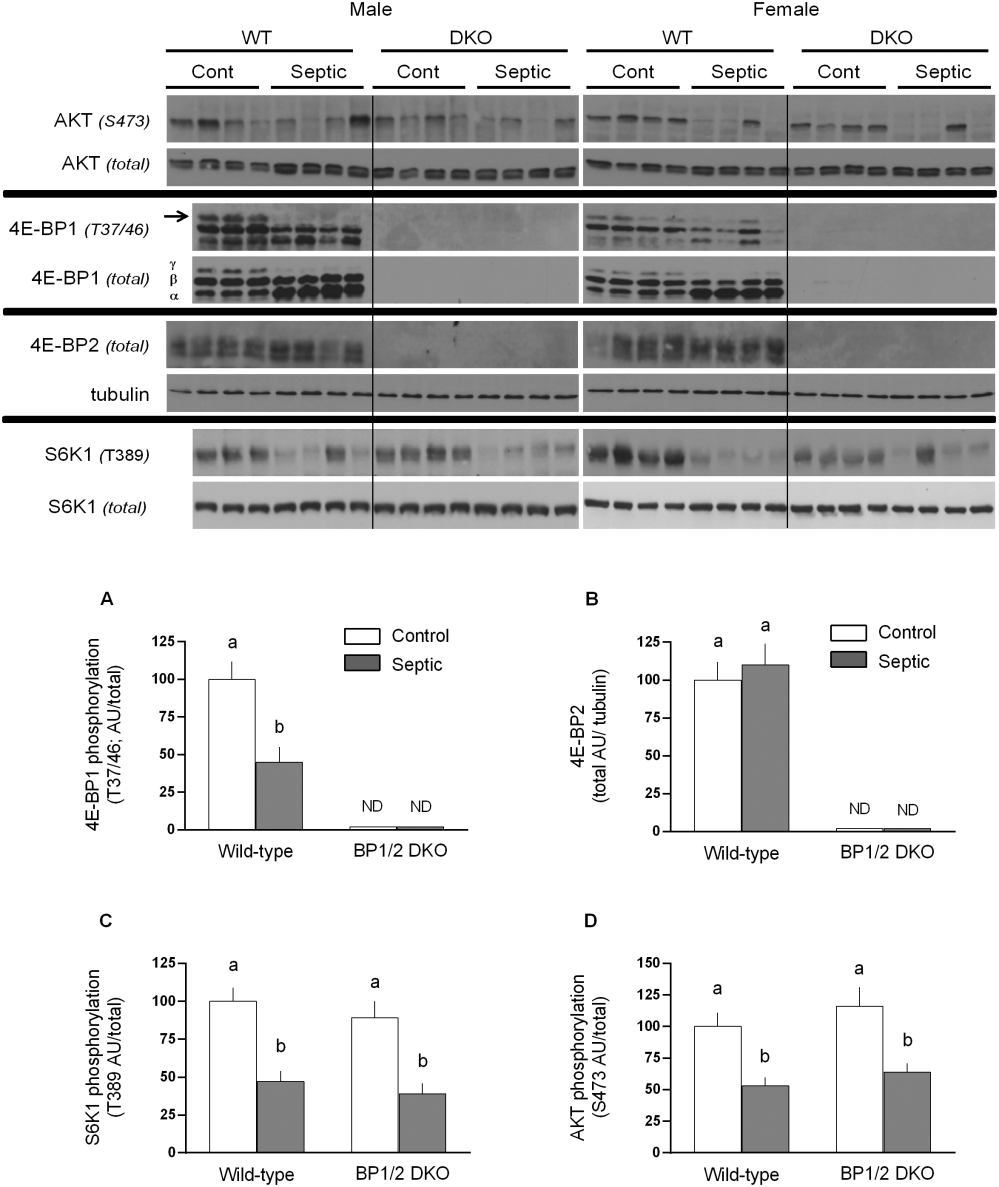
Representative Western blots for various proteins important in the control of muscle protein synthesis. Immunoblots for total and/or phosphorylated AKT, 4E-BP1, 4E-BP2, and S6K1 are presented for both male and female WT and 4E-BP1/BP2 DKO mice. Each lane represents a sample from a different mouse. Western blots were quantitated for male mice only, but data for female mice were qualitatively similar (data not shown). Bar graphs are means ± SEM; n = 8 male mice per group. For all bar graphs, values having a different superscript letter (a versus b) are statistically different (*P*<0.05); values with the same letter are not significantly different. ND  =  not detectable.

Protein synthesis is regulated in part by mTOR kinase which phosphorylates 4E-BP1 and S6K1 [8], [20]. As anticipated, both male and female DKO mice lacked detectable protein expression for both 4E-BP1 and 4E-BP2 in skeletal muscle (Figure 5) and liver (data not shown) by Western blot analysis. There was no compensatory increase in total or phosphorylated S6K1 under basal conditions or its downstream substrate ribosomal protein S6 (data not shown) in either male or female DKO mice compared to WT littermates. The amount of total and phosphorylated AKT, an upstream regulator of mTOR, did not differ between WT and DKO mice under nonseptic control conditions. Sepsis decreased phosphorylation of 4E-BP1, S6K1 and AKT by ∼50% in male WT mice. The sepsis-induced reduction in AKT and S6K1 phosphorylation did not differ between WT and DKO mice (Figure 5). Attempts to detect 4E-BP3 by Western blotting using commercial antibodies (Santa Cruz Biotechnology; Santa Cruz, CA, sc-162464) were not successful (data not shown).

**Figure 6:**
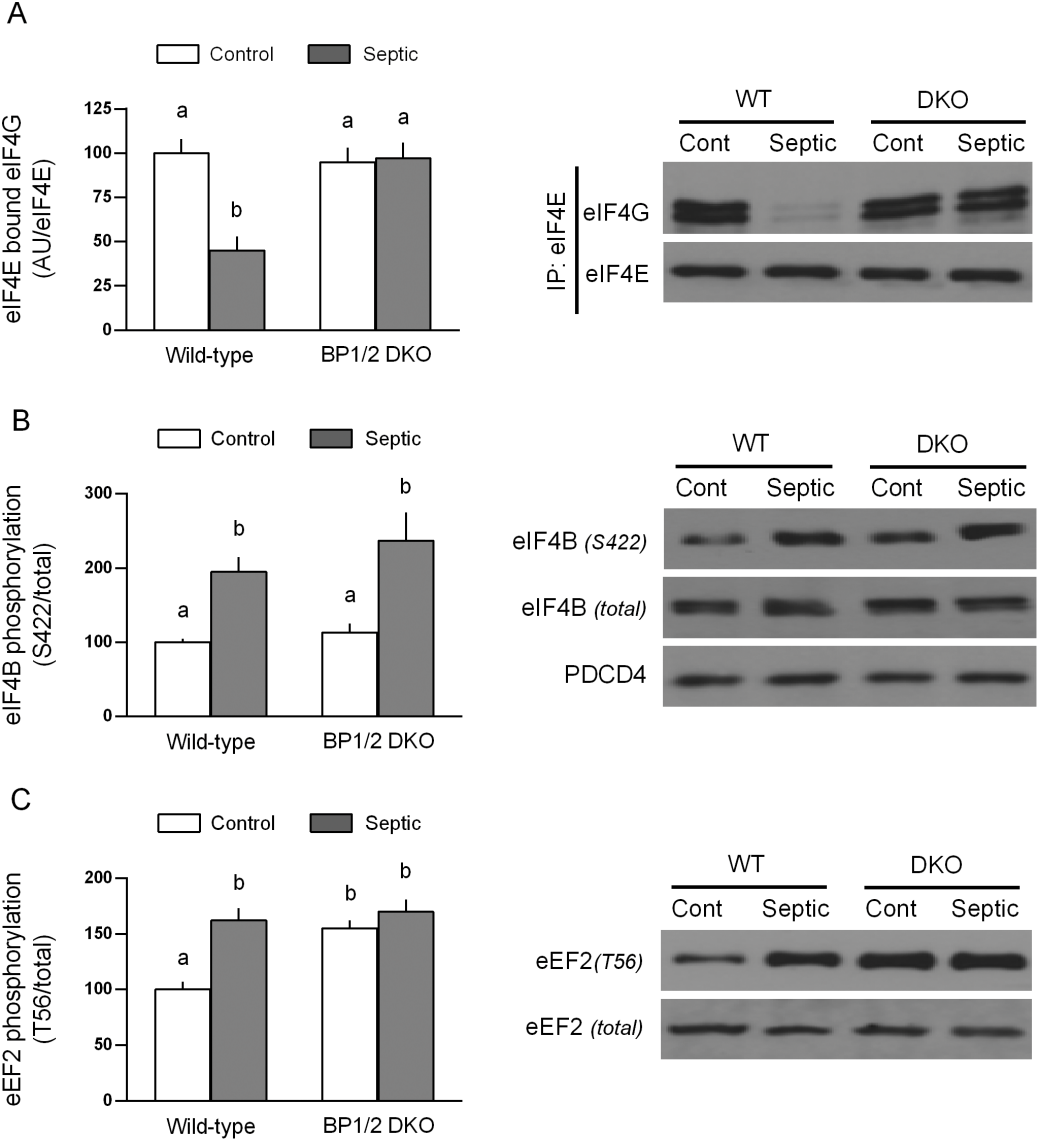
Amount of eIF4E•eIF4G complex as well as eIF4B and eEF2 phosphorylation in muscle from male WT and 4E-BP1/BP2 DKO mice under basal and septic conditions. Bar graphs are means ± SEM; n = 5 male mice per group (panel A) and 8–9 mice per group (panels B and C). For all bar graphs, values having a different superscript letter (a versus b) are statistically different (*P*<0.05); values with the same letter are not significantly different.

The extent of 4E-BP1 and 4E-BP2 phosphorylation regulates binding of eIF4G with eIF4E, thereby regulating cap-dependent translation [35]. While sepsis did not alter the total amount of eIF4E in muscle (data not shown), the amount of eIF4G bound to eIF4E in WT male mice was reduced 55% (Figure 6A). In contrast, the extent of eIF4E•eIF4G binding in DKO mice did not differ from WT values under either control conditions or in response to sepsis. Cap-dependent translation can also be controlled by eIF4B phosphorylation and PDCD4 [36]. The phosphorylation of eIF4B under basal conditions did not differ between WT and DKO mice, and both genotypes showed a comparable sepsis-induced increase in eIF4B phosphorylation (Figure 6B). The total amount PDCD4 was not altered in DKO mice or in response to sepsis (Figure 6B). Finally, mTOR can also regulate eEF2 phosphorylation which is inversely proportional to translation elongation [37]. Sepsis increased eEF2 phosphorylation 60% in muscle from WT mice (Figure 6C). Under basal control conditions, eEF2 phosphorylation was also increased approximately 60% in DKO mice, compared to WT animals. However, in contrast to WT mice, sepsis did not further increase eEF2 phosphorylation in DKO mice. As a result the extent of eEF2 phosphorylation did not differ between control and septic DKO mice.

### Protein Degradation

**Figure 7:**
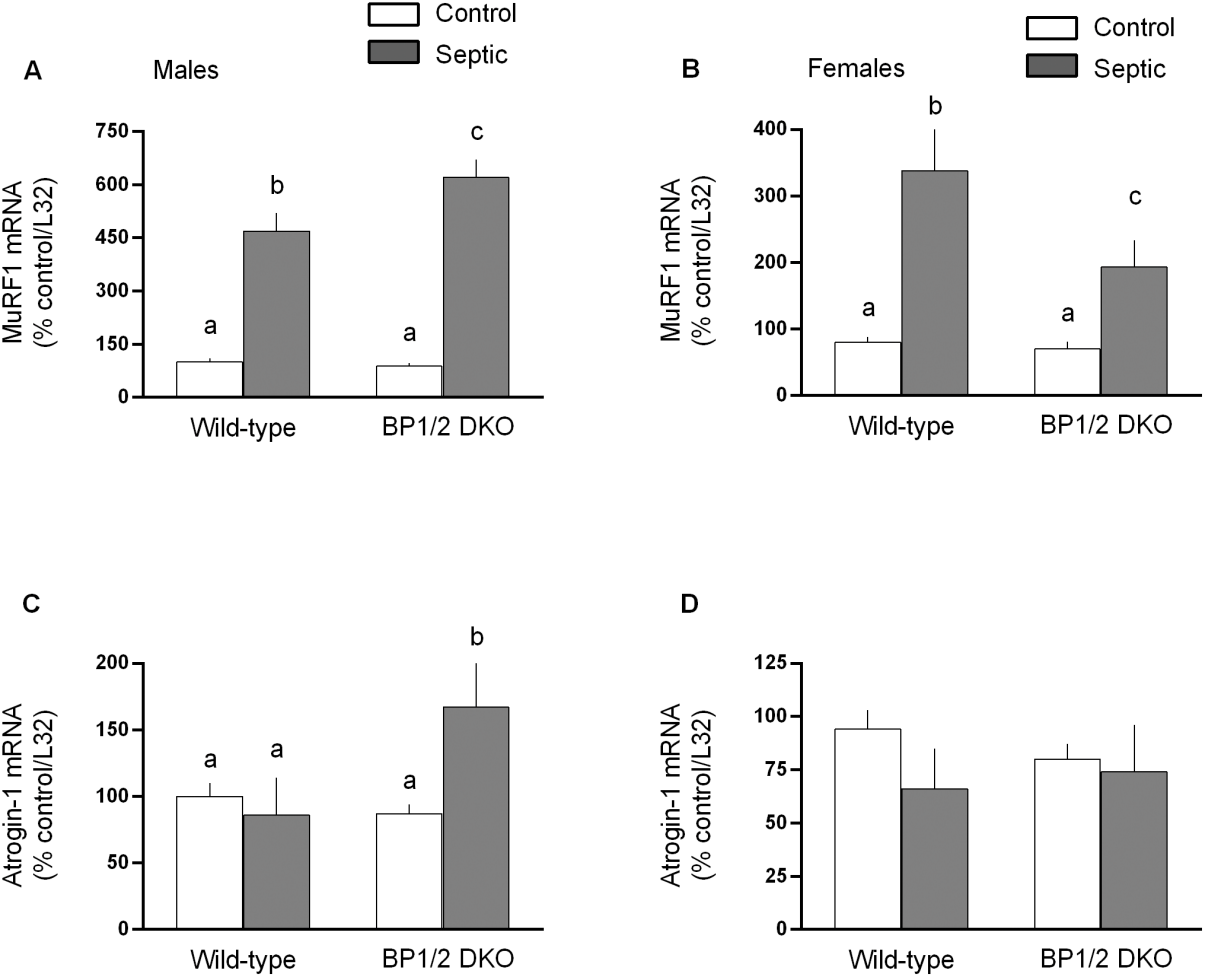
Sepsis-induced changes in the muscle-specific ubiquitin E3 ligases MuRF1 and atrogin-1 mRNA in gastrocnemius from male and female WT and 4E-BP1/BP2 DKO mice. Values are means ± SEM; n = 9–12 per group. Values are expressed as percent of control normalized for L32, where the male WT control value is arbitrarily set at 100%. For all bar graphs, values having a different superscript letter (a versus b versus c) are statistically different (*P*<0.05); values with the same letter are not significantly different.

Activation of the ubiquitin-proteasome pathway appears in part responsible for sepsis-induced muscle wasting [7], [19]. Therefore, we examined the muscle mRNA content for the muscle-specific E3 ligases MuRF1 and atrogin-1, as these “atrogenes” are coordinately up-regulated in a number of catabolic conditions [38]. However, sepsis only increased MuRF1 in both male and female WT mice (Figure 7A and 7B, respectively). The sepsis-induced increase in MuRF1 in male DKO mice was greater than that seen in WT males. Conversely, the sepsis-induced increase in MuRF1 in female mice DKO was smaller than in WT female control values. In contradistinction, we detected no sepsis-induced increase in atrogin-1 for WT male or female mice (Figure 7C and 7D, respectively). However, there was a sex effect in DKO mice, with only males exhibiting an increase in atrogin-1 in response to sepsis.

### Potential Regulators of Protein Balance

**Figure 8:**
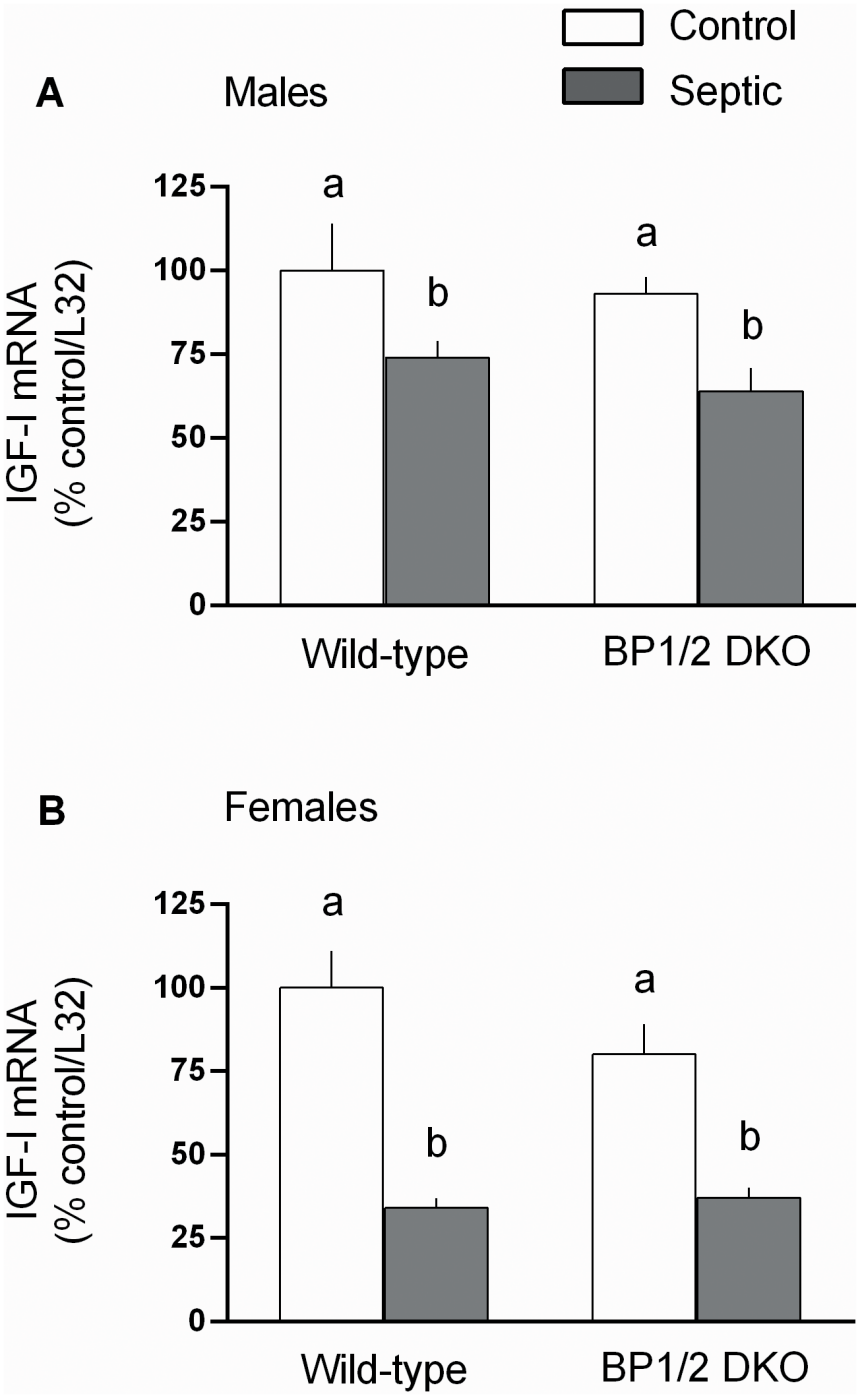
Sepsis-induced changes in the insulin-like growth factor (IGF)-I mRNA in gastrocnemius from male and female WT and 4E-BP1/BP2 DKO mice. Values are means ± SEM; n = 9–12 per group. Values are expressed as percent of control normalized for L32, where the male WT control value is arbitrarily set at 100%. For all bar graphs, values having a different superscript letter (a versus b) are statistically different (*P*<0.05); values with the same letter are not significantly different.

**Figure 9:**
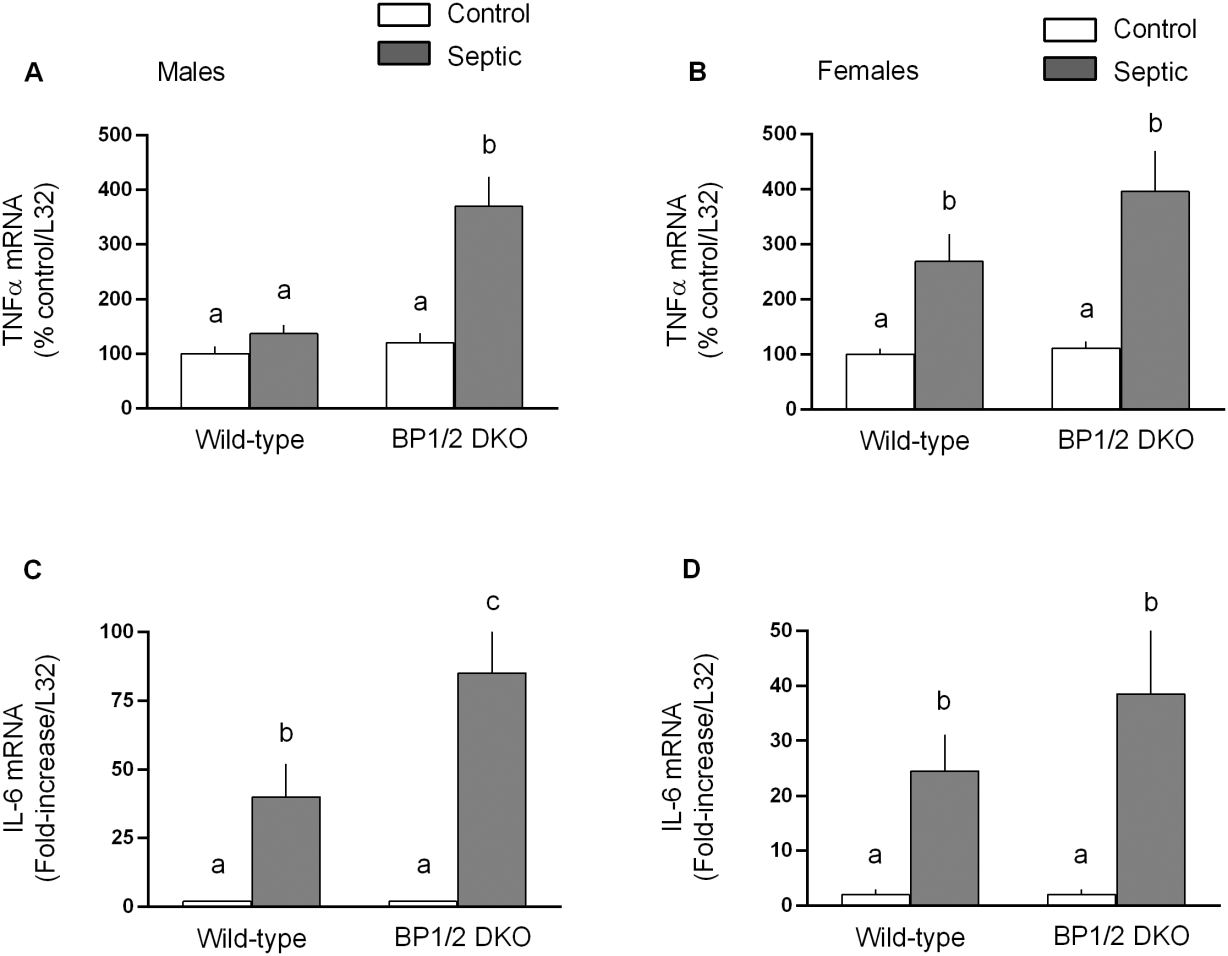
Sepsis-induced changes in tumor necrosis factor (TNF)-α and interleukin (IL)-6 mRNA content in gastrocnemius of in male and female WT and 4E-BP1/BP2 DKO mice. Values are means ± SEM; n = 9–12 per group. Values for TNFα are expressed as percent of control normalized for L32, where the male WT control value is arbitrarily set at 100%. For IL-6, values are expressed as fold-change above control values which was set at 1.0. For all bar graphs, values having a different superscript letter (a versus b versus c) are statistically different (*P*<0.05); values with the same letter are not significantly different.

Local and systemic concentrations of IGF-I are central in maintaining muscle protein balance [39] by effecting both rates of synthesis and degradation [40]. Therefore, IGF-I mRNA was examined as a possible mediator of the protein metabolic effects. Figure 8 illustrates sepsis decreased IGF-I mRNA in both male and female mice, but the reduction was greater (*P*<0.05) in both WT and DKO female mice. Elevations in inflammatory cytokines can contribute to muscle catabolism and inhibit mTOR [41]. In male mice, sepsis only increased muscle TNFα mRNA in DKO mice, not WT animals (Figure 9A), whereas sepsis increased TNFα in both genotypes of female mice (Figure 9B). A sepsis-induced increase in muscle IL-6 mRNA was detected in both male and female WT and DKO mice (Figure 9C and 9D), with the increase being greater in male DKO mice.

## Discussion

Using 4E-BP1/BP2 DKO mice, the present study investigated the potential in vivo regulatory role of these proteins in skeletal muscle protein balance under basal conditions and in response to a catabolic stress produced by sepsis. Our initial studies indicated there was no difference in body weight, LBM or fat mass between male and female DKO and WT at 1–4 months of age. Although at 1 year of age, both sexes had a small (<1%) but significant increase in fat mass with no change in LBM or total body weight. In contrast, 4E-BP1/BP2 DKO mice have been previously reported to exhibit an obese phenotype characterized by increased body weight and adipocyte size and mass [22]. Furthermore, this prior study also reported a higher RQ and lower rate of oxygen consumption in DKO mice, compared to WT controls, while RER, oxygen consumption and heat production did not differ between WT and DKO mice in our current study. The reason for these discrepant metabolic responses between studies is not readily apparent. While DKO mice were bred on the same genetic background, the macronutrient composition (percent protein/fat/carbohydrate of total calories) of the rodent chow provide in our study (23%/22%/55%) did differ from that provided in the original characterization of the DKO mice (20%/10%/70%). In contradistinction to both this early work and our study, fat mass was *reduced* and oxygen consumption *increased* in mice deficient in only 4E-BP1 [23] making the interpretation of data across studies uncertain.

Despite the normal growth and accretion of LBM in DKO mice, we posited that under basal conditions DKO mice had established a new homeostatic set-point where the reduction in muscle protein synthesis was counterbalanced by a proportional reduction in protein degradation. However, contrary to expectations, the in vivo-determined rate of basal protein synthesis in skeletal muscle did not differ between WT and DKO mice. The maintenance of muscle protein synthesis was surprising because of the absence of 4E-BP1 and 4E-BP2 proteins as well as the lack of a compensatory response in S6K1, which represents the other arm of the canonical mTORC1 signaling pathway. Moreover, the basal protein synthetic rate was also unchanged in liver and heart. At least for muscle, the maintenance of normal basal protein synthesis in DKO mice may be explained by the apparently normal binding of eIF4G with eIF4E. However, why eIF4G•eIF4E binding was not altered in DKO mice completely lacking the normally inhibitory eIF4E binding proteins is unknown [35]. On the other side of the protein balance equation, we also did not detect any genotype difference in the mRNA content for the muscle-specific ubiquitin E3 ligases, MuRF1 and atrogin-1. While we acknowledge in vivo proteolysis was not directly assessed in this study, elevations in mRNA for these two “atrogenes” have been reported in a diverse array of conditions associated with increased protein breakdown via activation of the ubiquitin-proteasome pathway and muscle wasting [32], [38], including sepsis [19], [42]. However, our investigation of protein degradation was limited in scope and therefore we cannot exclude the possibility that other pathways for breakdown (e.g., autophagy, apoptosis) were differentially regulated in WT and DKO mice either under control or septic conditions. Therefore, collectively our data suggest the rate of basal protein synthesis in skeletal muscle (and possibly other tissues) was unchanged in the DKO mice under basal conditions.

Next, based on maintenance of eIF4G association with eIF4E, we speculated the decrease in protein synthesis in muscle and other peripheral organs in response to a catabolic stimulus would be ameliorated in 4E-BP1/BP2 knockout mice as the lack of 4E-binding proteins would enhance cap-dependent translation initiation. However, again, our data did not support this hypothesis. Sepsis, produced by the well-characterized and validated model of CLP, produced many of the expected changes observed in humans [8], [19], including: reduction in total body weight, decreased skeletal muscle and liver protein synthesis, decreased 4E-BP1 and S6K1 phosphorylation in muscle, increased MuRF1 and/or atrogin-1, increased IL-6 and/or TNFα mRNA, and decreased in IGF-I mRNA in muscle. We speculate that these sepsis-induced changes were not of sufficient duration to produce a reduction in the mass of the gastrocnemius or quadriceps between control and septic mice. While some genotype effects were detected (i.e., larger increases in MuRF1, atrogin-1, IL-6 and TNFα in skeletal muscle of male DKO mice), there was no difference between WT and DKO animals in regards to their sepsis-induced decrease in body weight, acute 24-h survival, or the reduction in protein synthesis in muscle, heart or liver which might have been predicted given the exaggerated inflammatory cytokine response [8]. The up-regulated septic cytokine response in DKO mice would be consistent with the maintenance of eIF4E•eIF4G binding and mTOR activity in immune cells which might be expected to increase cytokine production [43].

For skeletal muscle, the similar decrease in protein synthesis for both septic WT and DKO mice is internally consistent with the comparable reduction in S6K1 (and S6, data not shown) phosphorylation. While sepsis did not alter the total amount of the m^7^GTP cap binding protein, eIF4E, it markedly decreased its interaction with the scaffolding protein eIF4G in WT mice. Such a decrease would be expected to reduce the binding of mRNA to the 43S preinitiation complex and thereby impair protein synthesis. In contradistinction, there was no sepsis-induced decrease in the amount of active eIF4E•eIF4G complex in muscle from DKO mice, although global protein synthesis was decreased. Collectively, while these data indicate 4E-BP1 phosphorylation and the interaction of eIF4E with eIF4G represent a regulatory control point under normal conditions (e.g., in WT mice), they also imply there must be at least one more additional control point distal to mRNA•eIF4E•eIF4G complex formation regulating the sepsis-induced decreases in muscle protein synthesis in the DKO mice.

In this regard, it has recently been reported that while eIF4E•eIF4G association is a necessary step in cap-dependent translation it is not sufficient for maximal stimulation of hepatic protein synthesis [36]. In this scenario, feeding stimulates S6K1 phosphorylation of both PDCD4 (releasing it from eIF4A and permitting eIF4A binding to eIF4G) and eIF4B (which enhances the interaction of eIF4A with eIF4G) ultimately stimulating eIF4A helicase activity [44], [45]. However, in the current study, sepsis increased eIF4B phosphorylation (despite a reduction is S6K1 phosphorylation) in muscle and this increase was comparable between WT and DKO mice. Moreover, as total PDCD4 is rapidly degraded upon its release from eIF4A, the lack of a detectable change in total PDCD4 suggests an alteration in eIF4A helicase activity is an unlikely mechanism for the sepsis-induced reduction in protein synthesis in the presence of normal eIF4E•eIF4G binding. Finally, we examined eEF2 as enhanced T56-phosphorylation of eEF2, by eEF2 kinase, reduces the affinity of this factor for the ribosome thereby inhibiting the translocation step of peptide-chain elongation [37]. The sepsis-induced increase in eEF2 phosphorylation in muscle of WT mice was consistent with the reduction in mTOR activity and global protein synthesis observed in these animals. However, a comparable increase in eEF2 phosphorylation was also detected in DKO mice in the basal control condition where muscle protein synthesis did not differ between WT and DKO mice. Moreover, the extent of eEF2 phosphorylation did not differ between control and septic DKO mice. Hence, while these data suggest that an impaired rate of elongation may contribute to the sepsis-induced decrease in muscle protein synthesis in WT mice, such a mechanism does not appear operational in the sepsis-induced reduction in protein synthesis detected in DKO mice. Furthermore, eEF2 phosphorylation was increased in DKO control mice, compared to WT controls, with no apparent reduction in global protein synthesis. Hence, the overall importance of eEF2 and elongation in regulating protein synthesis under these conditions appears unlikely and the exact locus for the regulatory step in cap-dependent mRNA translation in septic DKO mice remains to be determined.

In summary, our study demonstrates there are few overt differences during the first year of life between WT mice and those mice having a global deletion of both 4E-BP1 and -BP2. The rate of growth, whole-body energy production, and muscle weight do not differ between WT and DKO mice. The normal accretion of LBM in DKO mice was consistent with normal rates of protein synthesis in organs such as muscle, heart and liver as well as the apparently normal rate of muscle proteolysis. Moreover, deletion of 4E-BP1/BP2 did not alter mTOR signaling to S6K1 or the association of eIF4E•eIF4G in muscle under basal conditions. Additionally, DKO mice responded to sepsis with a catabolic response characterized by a reduction in muscle protein synthesis which was comparable in magnitude to that seen in WT mice. While sepsis decreased S6K1 phosphorylation in muscle from both WT and DKO mice, the sepsis-induced decrease in eIF4E•eIF4G binding seen in WT mice was absent in DKO mice. This latter finding suggests sepsis can down-regulate mRNA translation by a second mechanism distal to the formation of the 43S preinitiation complex.
